# Measurement invariance of the PHQ-9 and GAD-7 across males and females seeking treatment for common mental health disorders

**DOI:** 10.1186/s12888-023-04804-x

**Published:** 2023-04-28

**Authors:** Rob Saunders, Delilah Moinian, Joshua Stott, Henry Delamain, Syed Ali Naqvi, Satwant Singh, Jon Wheatley, Stephen Pilling, Joshua E.J. Buckman

**Affiliations:** 1 CORE Data Lab, Centre for Outcomes Research and Effectiveness (CORE), Research Department of Clinical, Educational and Health Psychology, UCL, London, UK; 2 ADAPT Lab, Research Department of Clinical, Educational and Health Psychology, UCL, London, UK; 3 Barking & Dagenham and Havering IAPT Services, North East London NHS Foundation Trust, London, UK; 4 Waltham Forest Talking Therapies, North East London NHS Foundation Trust, London, UK; 5 Talk Changes: City & Hackney IAPT Service, Homerton University Hospital NHS Foundation Trust, London, UK; 6 Camden and Islington NHS Foundation Trust, London, UK; 7 iCope - Camden and Islington Psychological Therapies Services, Camden & Islington NHS Foundation Trust, London, UK

**Keywords:** Measurement invariance, Gender, PHQ-9, GAD-7, Community mental health services

## Abstract

**Background:**

The nine-item Patient Health Questionnaire (PHQ-9) and the seven-item Generalised Anxiety Disorder scale (GAD-7) are routinely used in research and clinical practice. Whilst measurement invariance of these measures across gender has been demonstrated individually in general population studies and clinical samples, less is known about invariance of the distinct but correlated latent factors (‘depression’ and ‘anxiety’). The current study assessed measurement invariance of these constructs across males and females seeking treatment for common mental health disorders.

**Methods:**

Data were provided from eight psychological treatment services in London, England. Data from initial assessments with the services where individual items on the PHQ-9 and GAD-7 were available were included in analyses. Measurement invariance was explored across self-identified genders, with ‘male’ and ‘female’ categories available in the dataset. Sensitivity analyses were conducted using propensity score matching on sociodemographic and clinical variables.

**Results:**

Data were available for 165,872 patients (110,833 females, 55,039 males). There was evidence of measurement invariance between males and females in both the full sample and a propensity score matched sample (n = 46,249 in each group).

**Conclusions:**

Measurement invariance of the correlated depression and anxiety factors of PHQ-9 and GAD-7 were indicated in this sample of individuals seeking psychological treatment for CMHDs. These results support the use of these measures in routine clinical practice for both males and females. This is of particular importance for assessing the prevalence of clinically significant levels of symptoms as well as comparing treatment outcomes across genders.

**Supplementary Information:**

The online version contains supplementary material available at 10.1186/s12888-023-04804-x.

## Background

Common mental health disorders (CMHDs), including depression and anxiety disorders, affect hundreds of millions of people around the world each year [1]. Psychological therapies can be effective, but only around half of all patients recover by the end of their treatment [2, 3]. Efforts to improve treatment outcomes rely on robust means of measuring and monitoring symptom change, that need to be consistent and translatable across major patient subgroups who seemingly experience different clinical outcomes [4, 5]. The prevalence of CMHDs is typically higher in females than males, and females make up approximately two-thirds of the population of adults in receipt of treatments for CMHDs [6]. Several potential hypotheses have been suggested for differences in CMHD prevalence between males and females, but little attention has been given to the ways in which the symptoms of CMHDs are experienced between these groups. If there were differences in these experiences, the resultant measurement error would make it challenging to draw valid comparisons across males and females when using the same measurement tools or scales [4]. This might cast doubt on findings as to prevalence differences, but also on evidence suggesting no difference exists in treatment outcomes across males and females for people with depression [7], and have broader implications for the use of measures when monitoring and evaluating the effects of treatments. Two of the most commonly used measures of CMHD symptoms, the 9-item Patient Health Questionnaire (PHQ-9 [8]) and the 7-item Generalised Anxiety Disorder scale (GAD-7 [9]), are widely used in research and clinical practice. Thus, if it were found that these measures needed to be interpreted differently between males and females then this would have major consequences. Therefore, ascertaining measurement invariance in the use of these measures between males and females is important.

There have been several studies exploring psychometric differences in measures of depression and anxiety between males and females, specifically using the PHQ-9 and the GAD-7. General population studies have demonstrated measurement invariance in the PHQ-9 [4, 10, 11] and the GAD-7 [12, 13] in non-clinical samples between a range of sociodemographic groups, including by gender. Similar findings have been shown in clinical samples [14, 15]. However, given the PHQ-9 and GAD-7 are frequently completed together, both in clinical practice as well as in research studies to estimate prevalence, considering invariance of the latent structure of models including the PHQ-9 and GAD-7 as separate but correlated constructs of ‘depression’ and ‘anxiety’ is likely to have greater utility and to be of greater relevance to routine clinical practice and research. Such invariance has been demonstrated between different countries in general population studies [16], but less is understand about potential differences in males and females on these correlated constructs.

The aim of the current study was to explore measurement invariance of the PHQ-9 and GAD-7 as correlated latent variables between males and females seeking psychological treatment for CMHDs. Propensity score matching, where females and males were matched on a range of sociodemographic factors, was performed to add to the robustness of findings by controlling for measured confounding factors.

## Method

### Participants

The analytic sample comprised all individuals referred to eight Improving Access to Psychological Therapies (IAPT) services that were members of the North and Central East London IAPT service Improvement and Research Network (NCEL IAPT SIRN) [17, 18]. IAPT services provide evidence-based psychological treatments for depression and anxiety disorders, across all regions in England using a stepped care model (see[19] for details about these services). Data were used from January 2011 up until August 2020. Only baseline scores, that is scores from the initial assessment with the services, were included in the current analysis. Participants were included if: they had item-level data available for the PHQ-9 and GAD-7 from their initial assessment appointment, they were aged 18 years or older and had data available about their gender. Individuals were excluded whose presenting problem, the clinical disorder that is to be the focus of treatment matched to ICD-10 codes [20], was for a disorder for which there was no established IAPT treatment protocol, such as schizophrenia or substance-misuse problems [19]. At the time of data collection by these services, the only available options for recording self-identified gender were ‘male’, ‘female’, ‘not known’ or ‘not specified’. Consequently, it was not possible to estimate invariance across all genders with only individuals who reported being ‘female’ or ‘male’ included, (the other two response categories treated as missing). It is acknowledged that the binary terms male and female, whilst commonplace in research, even in reporting of randomised controlled trials [7], are the only two self-identifying gender descriptors available in the dataset and do not fully encapsulate the range of ways in which individuals might choose describe their gender.

### Measures

#### Patient health questionnaire nine-item (PHQ-9; [8])

The PHQ-9 is a self-report measure consisting of nine items which approximately match the criteria for depression from the Diagnostic and Statistical Manual of Mental Disorders fourth edition (DSM-IV) [21]. The items include anhedonia, low mood, sleep, fatigue, appetite, low self-esteem, concentration, psychomotor disturbance, and suicidal ideation. Each item is scored 0 (“Not at all”) to 3 (“Nearly every day”), with total scores ranging from 0 to 27.

#### Generalised anxiety disorder scale seven-item (GAD-7; [9])

The GAD-7 is a self-report measure consisting of seven items that match many of the criteria for generalised anxiety disorder in DSM-IV. Items include nervousness, uncontrollable worrying, worrying about different things, issues relaxing, restlessness, irritability, and fear. As above, items are scored between 0 and 3, with total scores ranging from 0 to 21.

### Additional variables

At the initial assessment, patients completed additional questions covering a range of sociodemographic and clinical variables. These included questions on: age, gender, ethnicity, employment status, and whether they are taking psychotropic medications. The Index of Multiple Deprivation (IMD) was available for individuals [22], and was collapsed into quintiles, with ‘1’ indicating the most deprived areas and ‘5’ the least deprived. Further details on these variables and their categorisation are presented in Appendix A.

### Statistical analysis

#### Multiple group confirmatory factors analysis (MGCFA)

**Fig. 1:**
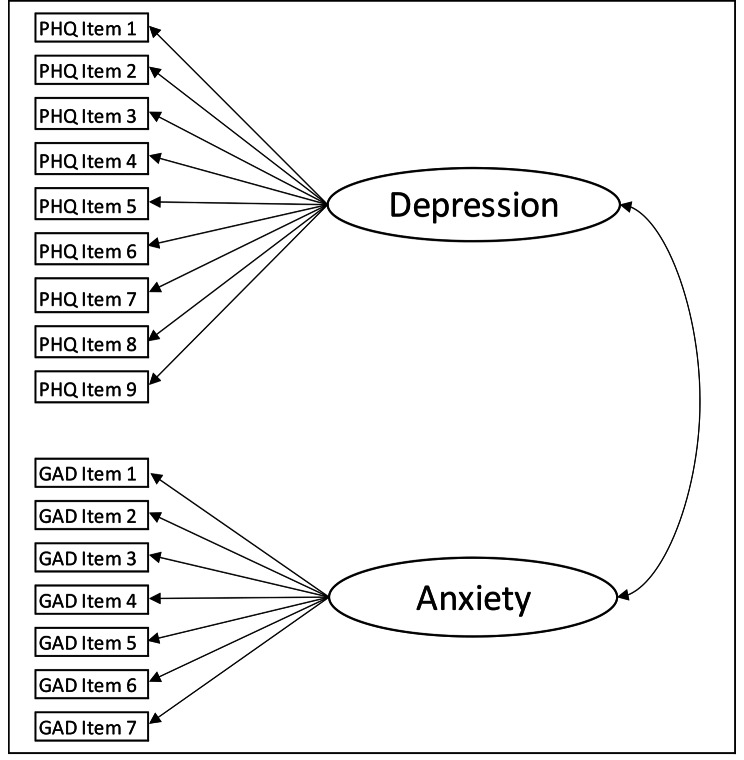
Structure of the proposed model

Whilst alternative factor structures of the PHQ-9 have been proposed, such as the Cognitive-Affective/Somatic structure [15], for the current analysis we considered only the unidimensional structure of each measure. This is because they are most commonly used in this manner in both research and clinical practice, and as correlations between the commonly identified sub-factors are high [15]. We first used confirmatory factor analysis (CFA) without considering gender groups to explore model fit. A model was estimated that used both the PHQ-9 and GAD-7 items, to construct two correlated latent variables for ‘depression’ and ‘anxiety’ respectively (see Fig. 1). Commonly used metrics of model fit for CFA were estimated: the comparative fit index (CFI), the root mean squared error of approximation (RMSEA) and the standardised root mean square residual (SRMR). CFI values of 0.90 and 0.95 are considered indicative of acceptable and good model fit respectively [23]. For the RMSEA we considered values < 0.05 indicative of close fit, 0.05–0.08 as acceptable fit and 0.08–0.1 as a moderate or ‘mediocre’ fit [24]. SRMR values below 0.05 are taken as good fit[23] and below 0.1 as acceptable [24].

Multiple group confirmatory factor analysis (MGCFA) was then conducted using genders. The following models of invariance were estimated:

- M1: Configural Invariance; same model structure between groups, and all parameters are free.
- M2: Metric Invariance; invariance in loadings between groups.
- M3: Scalar Invariance; invariance in loadings and intercepts.
- M4: Residual Invariance; invariance in loadings, intercepts and residuals.
- M5: Residual Invariance; invariance in loadings, intercepts, residuals and factor means.
- M6: Residual Invariance; invariance in loadings, intercepts, residuals, factor means and variances.

The decision on whether to adopt a model, whereby there was evidence of measurement invariance at that stage, was made by comparing the change in model fit statistics between the model in question (M) and the previous model (M-1). The change between models was considered within tolerated ranges, when the difference in the CFI value (ΔCFI) was < 0.01, the ΔRMSEA was < 0.015 and the ΔSRMR was < 0.030 [16, 25]. Whilst χ^2^ values were reported, they were not used to decide on model adoption due to known issues when using larger sample sizes [25].

#### Propensity score matching

Sensitivity analyses were conducted using a matched sample of males and females. Propensity score matching (PSM) methods [26] were used to explore whether measurement invariance was observed when the groups were balanced on measured sociodemographic variables. Matched controls for males (the smallest group) were identified in the sample of females, with the following matching variables: age, ethnicity, local healthcare trust, psychotropic medication status, employment status, IMD quintile and the year of the referral to the service. Any observation with missing data on matching variables was excluded. Baseline symptom severity (total scores of the PHQ-9 and GAD-7) was not included in the matching because this would artificially create similar group mean scores, potentially biasing the estimates of measurement variance. Problem descriptors were not included for the same reason, as these are likely to be related to symptom measure scores. Matching without replacement was performed that meant the same control individual could be used more than once, as the best match for more than one male observation as per previous analyses [27], with a narrow caliper of 0.0001 used. Once a matched control sample was identified, the MGCFA was repeated in the same procedure as described above for the full sample.

## Results

### Descriptive statistics

**Fig. 2:**
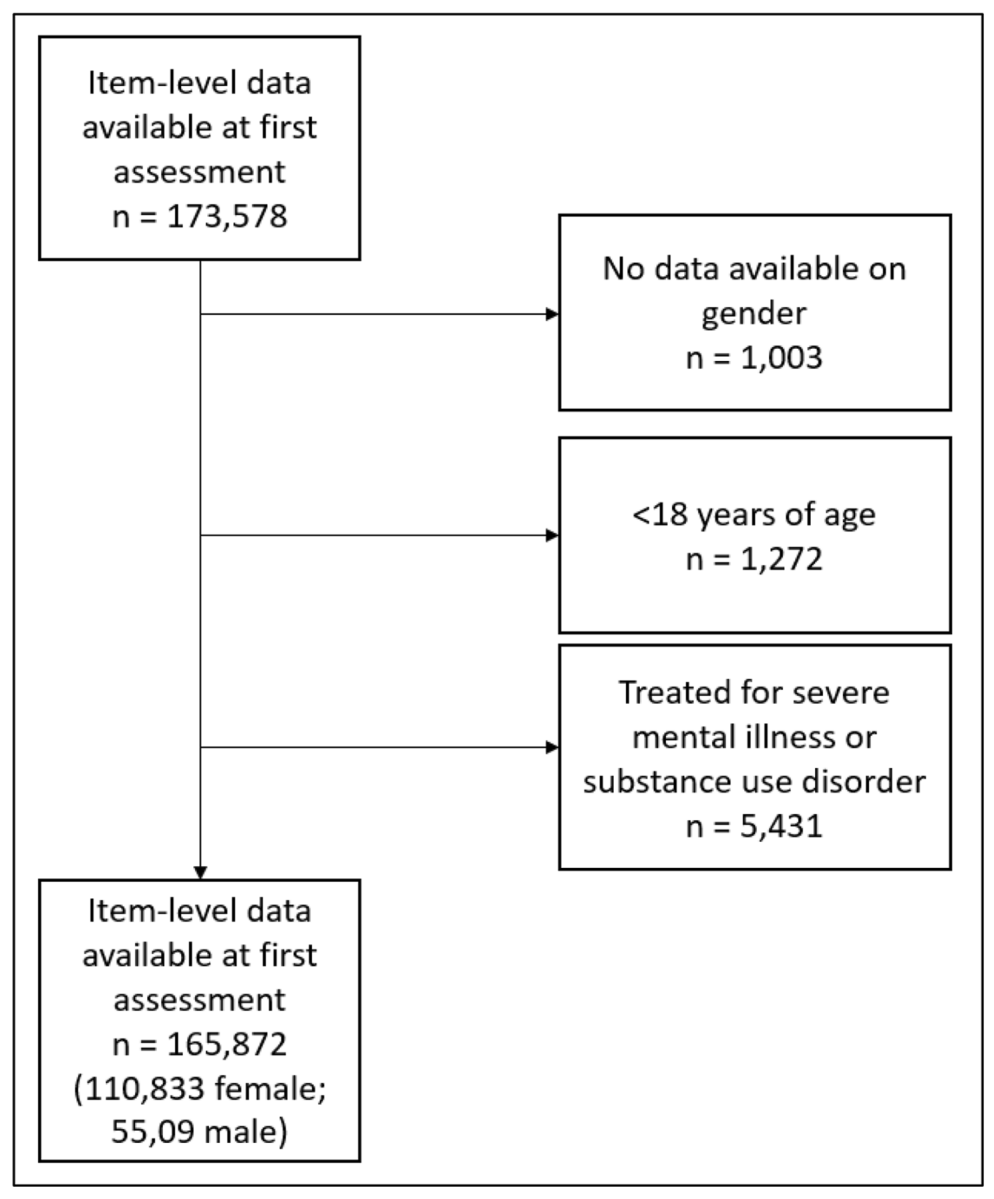
Patient flow diagram

From the initial dataset of 173,578 individuals with item-level data availble for the PHQ-9 and the GAD-7, n = 1,003 (0.58%) did not have a gender recorded (it was either missing or recorded as ‘not known’ or ‘prefer not to say’). In addition, n = 1,272 were less than 18 years old, and n = 5,431 were treated for a mental health condition for which there is not a treatment protocol in these services and were excluded (see Fig. 2 for patient flow diagram). The final analytic sample included n = 165,872 patients, where n = 110,833 (66.8%) were female and n = 55,039 (33.2%) were male. The sample gender split was representative of that observed in national evaluations of these services [28].

Descriptive statistics for the sample, split by gender, are presented in Table 1. There were differences in the distributions of all baseline variables between males and females although effect sizes for these differences were either small or negligible on recommended thresholds for Cramér’s V and Hedges’ g.

**Table 1:** Descriptive statistics and comparison split by gender

		Females		Males			
Variable	Category	N	%	N	%	P-value	Cramér’s V
Local Healthcare Organisation	Trust 1	27,324	24.65%	14,377	26.12%	< 0.001	0.022
	Trust 2	15,193	13.71%	7,993	14.52%		
	Trust 3	22,427	20.23%	10,758	19.55%		
	Trust 4	45,889	41.40%	21,911	39.81%		
Age	18–24	18,224	16.44%	7,496	13.62%	< 0.001	0.052
	25–34	38,241	34.50%	17,873	32.47%		
	35–44	23,385	21.10%	12,303	22.35%		
	45–54	16,832	15.19%	9,555	17.36%		
	55–64	9,218	8.32%	5,324	9.67%		
	65+	4,933	4.45%	2,488	4.52%		
Ethnicity	White	66,194	59.72%	33,977	61.73%	< 0.0001	0.047
	Asian	7,286	6.57%	2,935	5.33%		
	Black	12,102	10.92%	6,774	12.31%		
	Mixed	14,283	12.89%	5,783	10.51%		
	Other	5,561	5.02%	2,617	4.75%		
	Missing	5,407	4.88%	2,953	5.37%		
Psychotropic Medication	Not Taking	65,927	59.48%	31,634	57.48%	< 0.001	0.020
	Taking	35,998	32.48%	18,879	34.30%		
	Missing	8,908	8.04%	4,526	8.22%		
Employment status	Employed	80,074	72.25%	37,119	67.44%	< 0.001	0.052
	Unemployed	26,963	24.33%	16,041	29.14%		
	Missing	3,796	3.42%	1,879	3.41%		
IMD Quintile	1	38,745	34.96%	18,981	34.49%	0.003	0.011
	2	37,918	34.21%	18,724	34.02%		
	3	18,835	16.99%	9,428	17.13%		
	4	10,974	9.90%	5,560	10.10%		
	5	2,886	2.60%	1,497	2.72%		
	Missing	1,475	1.33%	849	1.54%		
Problem Descriptor	Depression	44,684	40.32%	22,409	40.71%	< 0.001	0.061
	Mixed A + D	6,474	5.84%	2,798	5.08%		
	GAD	15,518	14.00%	6,179	11.23%		
	OCD	1,596	1.44%	926	1.68%		
	PTSD	3,491	3.15%	1,895	3.44%		
	Social Phobia	1,982	1.79%	1,745	3.17%		
	Other phobia & panic	5,087	4.59%	2,377	4.32%		
	Unspecified anxiety	700	0.63%	360	0.65%		
	Not specified	31,301	28.24%	16,350	29.71%		
		Mean	SD	Mean	SD	P-value	Hedge’s g
PHQ-9 score at assessment		14.67	6.36	14.58	6.59	0.010	0.014
GAD-7 score at assessment		13.29	5.27	12.86	5.48	< 0.001	0.081

Notes. Mixed A + D = Mixed anxiety and depression

### Confirmatory factor analysis

CFA was initially conducted on the full sample and then in groups stratified by gender, before the main MGCFAs were performed. The full sample model demonstrated acceptable fit on all metrics (RMSEA = 0.079, CFI = 0.907, SRMR = 0.049), and was similar for both the female and male subgroups (female: RMSEA = 0.080, CFI = 0.903, SRMR = 0.050; male: RMSEA = 0.077 CFI = 0.917, SRMR = 0.046). Unidimensionality of the PHQ-9 and the GAD-7 was therefore indicated within the model.

### Multiple group confirmatory factor analysis – full sample

MGCFA results are presented in Table 2. The change in model fit statistics were below criteria values throughout the increasingly strict measurement invariance testing (from M1 to M6). The configural invariance model resulted in fit statistics similar to those presented for the full sample and all were within the acceptable range. There was limited change in fit statistics when examining the metric invariance model, suggesting metric invariance was achieved and that loadings were similar between genders. In the next stage (scalar invariance), there was no observed change in RMSEA and SRMR values, but the CFI decreased by 0.006. Whilst this was below the 0.01 critical value and therefore indicating scalar invariance was achieved, it hints that the intercepts between females and males are slightly different, even if this margin is very small. Models estimated for residual invariance, including with factor means and factor variances, all indicated measurement invariance.

**Table 2:** Multiple-group CFA and fit indices (full sample)

Model	χ2	df	CFI	RMSEA	SRMR	ΔCFI	ΔRMSEA	ΔSRMR
M1: Configural Invariance	106,206	206	0.908	0.079	0.048	--	--	--
M2: Metric Invariance	106,448	220	0.908	0.076	0.049	0.000	-0.003	0.001
M3: Scalar Invariance	112,671	234	0.902	0.076	0.049	-0.006	0.000	0.000
M4: Residual Invariance	113,343	250	0.902	0.074	0.049	0.000	-0.002	0.000
M5: M4 + factor means	113,960	252	0.901	0.074	0.049	-0.001	0.000	0.000
M6: M5 + factor variances	114,191	255	0.901	0.073	0.055	0.000	-0.001	0.006

### MGCFA – matched sample

Propensity score matching was then performed to create a matched sample of females and males. Only individuals with complete data on covariates were included in these analyses, resulting in n = 16,814 (15.17%) females and n = 8,770 (15.93%) males being excluded. From a sample of n = 46,269 males, matches could not be identified for n = 20 (0.04%), so they were excluded from further analyses. This resulted in n = 46,249 males and their matched controls being included in the sensitivity analyses. Sample comparison pre- and post-matching showed that whilst there were statistical differences between groups before matching, the matched groups were not statistically different on any of these variables after matching, and Cramer’s V values were all below 0.01 (see Appendix B).

Results of the MGCFA analyses with the propensity score matched sample, presented in Appendix C, indicated that the patterns of change between models were nearly identical to those for the analysis of the full sample. The biggest change was within the CFI value when estimating scalar invariance (ΔCFI=-0.005), but otherwise change values were within tolerated ranges, indicating measurement invariance of the correlated PHQ-9 and GAD-7 model between genders. It was also noted that the RMSEA and SRMR values were within the acceptable model fit range, but the CFI moved slightly below the critical value of 0.9 to 0.896 in M3 to M6.

## Discussion

This study observed measurement invariance of the correlated PHQ-9 and GAD-7 factors of depression and anxiety across males and females seeking psychological treatment for CMHDs. Unidimensionality of the PHQ-9 and the GAD-7 was indicated within the model. Measurement invariance was demonstrated in both the analysis of over 165,000 individuals, as well as analyses using a subset of males and females matched on sociodemographic variables (n = 92,249). Findings from this robust set of analyses suggest that these measures have utility in through assessing the same underlying construct (i.e., depression and anxiety) between males and females. This supports the use of these measures in clinical care, as well as in research for assessing symptoms of depression and anxiety and ascertaining likely prevalence. The model fit was just within the acceptable critical values based on conventional recommendations.

Measurement invariance was observed across groups and this finding supports previous research that has also identified invariance of these two measures in both the general population [4, 12] and in clinical samples [14, 15]. However, the fit of the current model was only just within acceptable limits according to standard recommendations for structural equation modelling, and poorer than the model fit statistics for the same model which explored measurement invariance of the PHQ-9 and GAD-7 separately between studies [16]. Comparing CFA fit of the studies using the general population compared to those using clinical samples suggests that poorer fit is often observed in unidimensional models of both the PHQ-9 and GAD-7 [4, 5, 14, 15, 29]. The current sample of patients assessed by IAPT services included those with a range of clinical disorders such as OCD, PTSD and panic disorder, and it may be the model structure differs between specific CMHDs, which warrants further investigation.

The MGCFA metrics demonstrated that no change in fit statistics exceeded the tolerated ranges, but it was noted that the CFI for the scalar model was greater than the change on any other value. Whilst still below threshold, indicating invariance between genders, it may suggest potential differences in scoring specific items between genders. Further exploration of item-by-gender differences may still be informative, despite measurement invariance being observed and other research suggesting a lack of differential item functioning by gender for the PHQ-9 and GAD-7 [16].

### Implications

This study has indicated measurement invariance of the PHQ-9 and GAD-7 across males and females, which supports the use of these scales to measure symptoms of depression and anxiety and make valid comparisons between these genders in clinical practice and research. This is particularly important given the routine use of these measures to estimate, for example, the prevalence of depression and anxiety in the general population, such as in surveillance studies [30] and in routine treatment settings for CMHDs [19], but also due to the importance of transdiagnostic assessment to inform treatment prognosis [31–33]. In these scenarios, where differences in either prevalence or levels of symptoms before and after treatment are regularly compared between males and females, unbiased measurement is a necessity [4]. As the PHQ-9 and GAD-7 are used as part of sessional outcome measurement in a large number of services across the world [19] demonstrating measurement invariance between groups is important for supporting clinical decision making, considering treatment progress, and to allow confidence in any relevant comparisons between male and female patients. However, given that the model fit statistics presented were on the limits of being considered acceptable, and lower than other studies using the same model structure [16], further research is needed to understand the latent structure of these models in individuals seeking treatment for CMHDs.

### Limitations

There are several limitations to the current analysis. At the time data were collected by the services, only binary self-identifying gender options were available which precluded the use of other categories of gender in the current analysis. It will be important to repeat these analyses to explore measurement invariance of these tools when data are available on sufficient numbers of participants who identify with other gender categories. The study included a large sample of participants, but all were drawn from services in London, England, and as other sources of variance may be under-represented here, generalisability to other locations or settings is unknown and may warrant further investigation, particularly if sources of measurement variance were shown to be socially or culturally bound and to act with cumulative effects. Propensity score matching was used to control for measured confounding factors but unmeasured and residual confounding cannot be ruled out. Further, although factorial invariance has been demonstrated when PHQ-9 and GAD-7 measures are completed face-to-face or over the telephone [34] this has not been demonstrated when additionally comparing measure scores collected via digital means, which are now the commonest way of completing the PHQ-9 and GAD-7 in IAPT services. Being able to discuss individual questions with clinicians or other staff at the services might have helped clarify items for some individuals, and the impact of this could be explored in future research.

## Conclusions

This study observed measurement invariance for the PHQ-9 and GAD-7 across males and females in a sample seeking psychological treatment for CMHDs. Findings were replicated for a propensity score matched sub-sample of 46,249 males and their matched controls. These results support the use of these measures in services to assess symptoms of depression and anxiety, to understand the need for treatment, and assess outcomes following intervention for both male and female patients, and that differences between males and females can be compared.

## Electronic supplementary material

Below is the link to the electronic supplementary material.

Supplementary Material 1

## Data Availability

The data that support the findings of this study were provided by the services as part of the NCEL agreement, but restrictions apply to the availability of these data, which were used under license for the current study, and so are not publicly available. Requests to access to the data can be made to the corresponding author (r.saunders@ucl.ac.uk), and permission would be agreed by individual services.
