# Supplementary material for: Measurement invariance of the PHQ-9 and GAD-7 across males and females seeking treatment for common mental health disorders
Source: BMC Psychiatry. 2023 Apr 28;23:298. doi: 10.1186/s12888-023-04804-x (PMC10148535; doi:10.1186/s12888-023-04804-x)
Supplement: Supplementary file 1 — Supplementary Material 1 [file 12888_2023_4804_MOESM1_ESM.docx]

Supplementary materials to:

**Measurement invariance of the PHQ-9 and GAD-7 across males and females seeking treatment for common mental health disorders**

**Content:**

**Appendix A: Sociodemographic variables and categories used (Page 2)**

**Appendix B: Comparison of pre and post matched samples (Page 3)**

**Appendix C: Multiple-group CFA and fit indices for the matched sample. (Page 5)**

**Appendix A: Sociodemographic variables and categories used.**

| **Variable** | **Description** | **Categories used in analysis** |
| --- | --- | --- |
| Gender | Self-identified gender descriptor | “Male”, “Female”, “Not known” or “Not specified”. |
| Local Healthcare organisation | Healthcare trust | "Trust 1", "Trust 2", "Trust 3" or "Trust 4". |
| Age | Self-reported age | Six categories: "18-24 years"; "25-34 years"; "45-54 years"; "55-64 years"; "65+ years old". |
| Ethnicity | Self-reported ethnicity | "White", "Asian", "Black", "Mixed", "Other ethnicity", based on ONS categories. |
| Psychotropic Medication | Psychotropic Medication status | Either "Not taking" or "Taking" psychotropic medication(s). |
| Employment | Employment status | Either "Employed" or "unemployed". |
| IMD Quintile | IMD rank collapsed into quintiles | Either “1”, “2”, “3”, “4” or “5”. |
| Year of referral | Referral year to the service | Either “2011”, “2012”, “2013, “2014”, “2015”, “2016”, “2017”, “2018”, “2019”, “2020”. |
| Problem descriptor | Description of the mental health condition treated | "Depression", "Mixed anxiety and depression", "Generalised anxiety disorder (GAD)", "Obsessive-compulsive disorder (OCD)", "Post-traumatic stress disorder (PTSD)", "Social Phobia", "Other phobias and panic", "Unspecified anxiety" or "Not specified" in the records. |

**Appendix B: Comparison of pre and post matched samples**

Table B1 presents the descriptive statistics of females and males before matching, and indicates differences were observed on all variables to be used in matching except IMD quintile. Table B2 shows that the matched samples (following propensity score matching) were balanced, with no statistically significant differences observed across variables between the two groups.

**Table B1: Comparison of females and males before matching.**

|  |  | Females (n=94,019) | | Males (n=46,269) | |  |  |
| --- | --- | --- | --- | --- | --- | --- | --- |
| Variable | Category | N | % | N | % | P-value | Cramer's V |
| Local Healthcare Organisation | Trust1 | 20,392 | 21.69% | 10,538 | 22.78% | <0.001 | 0.020 |
|  | Trust2 | 13,404 | 14.26% | 7,036 | 15.21% |  |  |
|  | Trust3 | 20,249 | 21.54% | 9,668 | 20.90% |  |  |
|  | Trust4 | 39,974 | 42.52% | 19,027 | 41.12% |  |  |
| Age | 18-24 | 15,764 | 16.77% | 6,395 | 13.82% | <0.001 | 0.054 |
|  | 25-34 | 32,653 | 34.73% | 15,116 | 32.67% |  |  |
|  | 35-44 | 19,747 | 21.00% | 10,420 | 22.52% |  |  |
|  | 45-54 | 14,103 | 15.00% | 7,989 | 17.27% |  |  |
|  | 55-64 | 7,720 | 8.21% | 4,400 | 9.51% |  |  |
|  | 65+ | 4,032 | 4.29% | 1,949 | 4.21% |  |  |
| Ethnicity | White | 59,382 | 63.16% | 30,324 | 65.54% | <0.001 | 0.049 |
|  | Asian | 6,491 | 6.90% | 2,604 | 5.63% |  |  |
|  | Black | 10,816 | 11.50% | 6,072 | 13.12% |  |  |
|  | Mixed | 12,387 | 13.17% | 4,928 | 10.65% |  |  |
|  | Other | 4,943 | 5.26% | 2,341 | 5.06% |  |  |
| Psychotropic Medication | Not Taking | 60,761 | 64.63% | 28,983 | 62.64% | <0.001 | 0.050 |
|  | Taking | 33,258 | 35.37% | 17,286 | 37.36% |  |  |
| Employment status | Employed | 70,583 | 75.07% | 32,563 | 70.38% | <0.001 | 0.019 |
|  | Unemployed | 23,436 | 24.93% | 13,706 | 29.62% |  |  |
| IMD Quintile | 1 | 32,439 | 34.50% | 15,792 | 34.13% | 0.2 | 0.007 |
|  | 2 | 32,618 | 34.69% | 15,939 | 34.45% |  |  |
|  | 3 | 16,560 | 17.61% | 8,275 | 17.88% |  |  |
|  | 4 | 9,812 | 10.44% | 4,940 | 10.68% |  |  |
|  | 5 | 2,590 | 2.75% | 1,323 | 2.86% |  |  |
| Referral Year | 2011 | 2,843 | 3.02% | 1,483 | 3.21% | <0.001 | 0.0179 |
|  | 2012 | 4,772 | 5.08% | 2,551 | 5.51% |  |  |
|  | 2013 | 6,943 | 7.38% | 3,526 | 7.62% |  |  |
|  | 2014 | 8,468 | 9.01% | 4,321 | 9.34% |  |  |
|  | 2015 | 9,491 | 10.09% | 4,860 | 10.50% |  |  |
|  | 2016 | 11,599 | 12.34% | 5,697 | 12.31% |  |  |
|  | 2017 | 13,454 | 14.31% | 6,476 | 14.00% |  |  |
|  | 2018 | 15,647 | 16.64% | 7,611 | 16.45% |  |  |
|  | 2019 | 16,013 | 17.03% | 7,524 | 16.26% |  |  |
|  | 2020 | 4,789 | 5.09% | 2,220 | 4.80% |  |  |

**Table B2: Comparison of females and males following matching.**

|  |  | Females (n=46,249) | | Males (n=46,249) | |  |  |
| --- | --- | --- | --- | --- | --- | --- | --- |
| Variable | Category | N | % | N | % | P-value | Cramer's V |
| Local Healthcare Organisation | Trust1 | 10,553 | 22.82% | 10,528 | 22.76% | 0.901 | 0.003 |
|  | Trust2 | 6,945 | 15.02% | 7,028 | 15.20% |  |  |
|  | Trust3 | 9,690 | 20.95% | 9,667 | 20.90% |  |  |
|  | Trust4 | 19,061 | 41.21% | 19,026 | 41.14% |  |  |
| Age | 18-24 | 6,311 | 13.65% | 6,391 | 13.82% | 0.64 | 0.006 |
|  | 25-34 | 15,165 | 32.79% | 15,116 | 32.68% |  |  |
|  | 35-44 | 10,527 | 22.76% | 10,419 | 22.53% |  |  |
|  | 45-54 | 8,030 | 17.36% | 7,980 | 17.25% |  |  |
|  | 55-64 | 4,352 | 9.41% | 4,394 | 9.50% |  |  |
|  | 65+ | 1,864 | 4.03% | 1,949 | 4.21% |  |  |
| Ethnicity | White | 30,622 | 66.21% | 30,320 | 65.56% | 0.088 | 0.009 |
|  | Asian | 2,507 | 5.42% | 2,601 | 5.62% |  |  |
|  | Black | 6,030 | 13.04% | 6,060 | 13.10% |  |  |
|  | Mixed | 4,895 | 10.58% | 4,927 | 10.65% |  |  |
|  | Other | 2,195 | 4.75% | 2,341 | 5.06% |  |  |
| Psychotropic Medication | Not Taking | 29,121 | 62.97% | 28,972 | 62.64% | 0.311 | 0.003 |
|  | Taking | 17,128 | 37.03% | 17,277 | 37.36% |  |  |
| Employment status | Employed | 32,753 | 70.82% | 32,559 | 70.40% | 0.161 | 0.005 |
|  | Unemployed | 13,496 | 29.18% | 13,690 | 29.60% |  |  |
| IMD Quintile | 1 | 15,901 | 34.38% | 15,787 | 34.13% | 0.149 | 0.009 |
|  | 2 | 16,037 | 34.68% | 15,936 | 34.46% |  |  |
|  | 3 | 8,258 | 17.86% | 8,270 | 17.88% |  |  |
|  | 4 | 4,847 | 10.48% | 4,935 | 10.67% |  |  |
|  | 5 | 1,206 | 2.61% | 1,321 | 2.86% |  |  |
| Referral Year | 2011 | 1,435 | 3.10% | 1,479 | 3.20% | 0.935 | 0.0062 |
|  | 2012 | 2,523 | 5.46% | 2,547 | 5.51% |  |  |
|  | 2013 | 3,526 | 7.62% | 3,524 | 7.62% |  |  |
|  | 2014 | 4,307 | 9.31% | 4,321 | 9.34% |  |  |
|  | 2015 | 4,838 | 10.46% | 4,859 | 10.51% |  |  |
|  | 2016 | 5,757 | 12.45% | 5,695 | 12.31% |  |  |
|  | 2017 | 6,566 | 14.20% | 6,475 | 14.00% |  |  |
|  | 2018 | 7,635 | 16.51% | 7,607 | 16.45% |  |  |
|  | 2019 | 7,530 | 16.28% | 7,523 | 16.27% |  |  |
|  | 2020 | 2,132 | 4.61% | 2,219 | 4.80% |  |  |

**Appendix C: Multiple-Ggroup CFA and fit indices for the matched sample.**

**Table C1.** **Multiple-group CFA and fit indices for the matched sample.**

| Model | χ2 | df | CFI | RMSEA | SRMR | ΔCFI | ΔRMSEA | ΔSRMR |
| --- | --- | --- | --- | --- | --- | --- | --- | --- |
| M1: Configural Invariance | 62286 | 206 | 0.904 | 0.081 | 0.050 | -- | -- | -- |
| M2: Metric Invariance | 62804 | 220 | 0.904 | 0.078 | 0.051 | 0.000 | -0.003 | 0.001 |
| M3: Scalar Invariance | 65938 | 234 | 0.899 | 0.078 | 0.052 | -0.005 | 0.000 | 0.001 |
| M4: Residual Invariance | 66663 | 250 | 0.898 | 0.076 | 0.052 | -0.001 | -0.002 | 0.000 |
| M5: M4+factor means | 67445 | 252 | 0.896 | 0.076 | 0.052 | -0.002 | 0.000 | 0.000 |
| M6: M5+factor variances | 67545 | 255 | 0.896 | 0.076 | 0.055 | 0.000 | 0.000 | 0.003 |
